# Genetic and phenotypic characterization of the novel mouse substrain C57BL/6N Korl with increased body weight

**DOI:** 10.1038/s41598-017-14196-0

**Published:** 2017-10-27

**Authors:** Kyung-Min Choi, Jaehoon Jung, Young Min Cho, Kwondo Kim, Mi-Gyeong Kim, Jinho Kim, Huibae Kim, Hee Jung Shin, Hae Deun Kim, Seung-Tae Chung, Seoae Cho, Myeon-Woo Chung

**Affiliations:** 1 Laboratory Animal Resources Division, Toxicological Evaluation and Research Department, National Institute of Food and Drug Safety Evaluation, Cheongju, 28159 Republic of Korea; 2 C&K Genomics, 26 Beobwon-ro 9-gil Bldg. C, #1008 (H business park) Songpa-gu, Seoul, 05836 Republic of Korea; 3 Interdisciplinary Program in Bioinformatics, Seoul National University, Kwan-ak St. 599, Kwan-ak Gu, Seoul, 151-741 Republic of Korea; 4 Department of Agricultural Biotechnology, Seoul National University, Kwan-ak St. 599, Kwan-ak Gu, Seoul, 151-742 Republic of Korea

## Abstract

In inbred mouse lines, there is generally little genetic difference between individuals. This small genetic variability facilitates carrying out research on minute changes of various traits and the gene pool. Also, characterizing the diversity and detecting selective genetic and phenotypic signatures are crucial to understanding the genomic basis of a population and to identify specific patterns of evolutionary change. In this study, we investigated the underlying genetic profiles of a newly developed mouse strain, C57BL/6NKorl (Korl), established through sibling mating over 30 generations. To analyse the distinctive genomic features of Korl mice, we used whole-genome sequencing from six samples, which were compared to those of other C57BL/6N-based mouse strains. Korl strain-specific polymorphisms were identified and signatures of a selective sweep were detected. In particular, the candidate genes related to the increased body weight of the Korl strain were identified. Establishment of the genetic profile of Korl mice can provide insight into the inbreeding-induced changes to the gene pool, and help to establish this strain as a useful model for practical and targeted research purposes.

## Introduction

New mouse strains are typically established by inbreeding to obtain models for particular research purposes. These inbred mice generally show almost identical genetic structures (up to 98% similarity) among individuals^1^. Such genetic homogeneity allows for researchers to obtain a more or less uniform response in a given model when conducting an experiment, and has advantages with respect to statistical reproducibility. In addition, the use of inbred mouse strains has contributed to gaining a better understanding of multiple biological mechanisms and metabolic pathways. However, despite the highly similar genetic structure among individuals, evident phenotypic^2^ and genetic variations (up to 2%) are apparent in inbred mice, even among littermates^3^. Therefore, investigations on the genetic diversity in inbred mice with similar genetic structures are important to gain a better understanding of the genomic basis of distinctive phenotypes.

C57BL/6N is one of the most popular strains of inbred mice. This strain is characterized by a small body and high reproductive performance, with a similar anatomy and physiology to humans, making it a useful vertebrate research model of human diseases. Accordingly, several substrains of C57BL/6N mice have been developed worldwide for various research purposes^4,5^. Although these substrains seem to show high genetic similarity, each strain has distinct phenotypic and genotypic characteristics. The strains have been sibling-mated (i.e. inbred) for over dozens of generations in different laboratories and institutes, and are now more than 20 generations apart. Therefore, despite having a common ancestor, these mice are expected to show phenotypic and/or genetic differences^4,6,7^. For instance, C57BL/6JOlaHsd is a substrain of C57BL/6J mice established through inbreeding with deletion of the *Snca* gene resulting in neurodegenerative disease^8^, but do not show a clear difference in phenotype from the parental strain. This strain provides a useful model for neuroscience research in gaining insight on the neuronal development and function of specific genes. As shown in this case, research with such models should be conducted after gaining a detailed understanding of the genetic properties that are unique to each strain so as to best delineate the mechanisms inducing the observed differences^9^.

A new inbred mouse strain, C57BL/6NKorl (Korl), was recently developed in the C57BL/6N background by the National Institute of Food and Drug Safety Evaluation, which shows distinctive characteristics, but these have not yet been reported or studied in detail. Korl mice have been sib-mated for over 33 generations since 2005 and have been managed exhaustively (see Supplementary Data S1). In the present study, we aimed to determine the characteristic features of the Korl strain with respect to its parental and control strains, and to elucidate the underlying genetic mechanisms contributing to these differences.

We investigated the general phenotypes of Korl mice, and performed whole-genome sequencing of the Korl strain and control strains, two originating from Taconic (Tac) and two from Charles River (Crl), to identify their genetic properties. Based on these genomic data, we analysed the genetic profiles of Korl strain-specific genetic regions and identified the candidate genomic characteristics associated with its traits. We expect that this information should provide insight for understanding C57BL/6N substrains at the genomic level, and promote their further development for specific research applications.

## Materials and Methods

### Animals

The protocols for the animal experiment were reviewed for ethical and scientific care procedures and were approved by NIFDS Institutional Animal Care and Use Committee (Approval Number: 1501MFDS-11). C57BL/6NKorl mice were housed in the laboratory animal facility of the NIFDS, and the other mice were purchased from commercial companies: Japan SLC (Shizuoka, Japan), Orient Bio (Gyeonggi-do, Korea), Daehan Biolink (Chungcheongbuk-do, Korea), and Koatech (Pyeongtaek, Korea). All mice were housed in specific pathogen-free conditions at 22 ± 1 °C under a relative humidity of 50 ± 10% with 12 h of light per day. All methods including housing the mice were performed in accordance with the Association for Assessment and Accreditation of Laboratory Animal Care International (AAALAC-I, Accredited Unit Number: 001492).

### Simple sequence length polymorphism analysis of microsatellites and polymerase chain reaction (PCR) of *Nnt*

To confirm genetic background of Korl mice, microsatellite marker analysis was performed using BALB/cCrSlc, DBA/2CrSlc, C57BL/6NCrSlc, and C57BL/6NKorl mice. DNA samples of the mice were extracted from the tail tissues. A total of 19 microsatellite markers were used according to a previous report^10^ following Harlan Company’s Genetic Monitoring Guideline for Inbred Mice & Rats. Primers for the markers are listed in Supplementary Table S1. PCR amplification was conducted with the following program: 94 °C for 5 min; 30 cycles of 94 °C for 1 min, 55 °C for 1 min, 72 °C for 30 s; and a final extension at 72 °C for 10 min. For confirmation of the presence of the nicotinamide nucleotide transhydrogenase (*Nnt*) gene, which is specifically deleted in C57BL/6J and not in C57BL/6N mice^11^, DNA samples were extracted from the tail tissues of mice belonging to the C57BL/6 strain (C57BL/6NCrSlc, C57BL/6NCrl, C57BL/6NHsd, C57BL/6NTac, C57BL/6JmSlc, and C57BL/6NKorl). Primers of exons in the *Nnt* gene were designed according to previous reports^4,11^ and are summarized in Supplementary Table S2. The PCR conditions for amplifying *Nnt* were as follows: 94 °C for 15 min; 34 cycles of 94 °C for 30 s, 61 °C for 90 s, 72 °C for 90 s; and a final extension at 72 °C for 10 min. After the PCR products were resolved using 4% agarose gels for 120 min at 75 V, microsatellites and the presence of the *Nnt* gene were analysed.

### Major histocompatibility complex (MHC) analysis

Typing of the MHC haplotype was performed as described previously^12^. In brief, the spleens of mice in the 16^th^ week were collected and homogenized. Microplates were coated overnight with 5 μg/mL goat anti-mouse IgG Fc (Sigma Aldrich, A0168) at 4 °C and then washed with phosphate buffered-saline (PBS)-Tween three times and blocked for 1 h in 3% bovine serum albumin at 37 °C. After washing again with PBS-Tween, the spleen homogenates from C57BL/6 mice were coated and incubated at 37 °C for 1 h. The blank sample was treated with PBS only, and spleen homogenates from BALB/c mice were used as a negative control. The plates were washed in PBS-Tween again, and were incubated with the following biotin-labelled antibodies (1 μg/mL) targeting each MHC haplotype at 4 °C for 30 min: H-2Kb (eBioscience, 11-5958-80), H-2Kd/H-2Dd (eBioscience, 11-5998-81), H-2Db (eBioscience, 11-5999-82), I-A/I-E (eBioscience, 13-5321-81), Igh-6a (BD Pharmingen, 553511), Igh-6b (BD Pharmingen, 553521), Igh-5a (BD Pharmingen, 553506), Igh-5b (BD Pharmingen, 553510), Igh-4a (BD Pharmingen, 553500), Igh-4b (BD Pharmingen, 553533), Igh-41a (BD Pharmingen, 553502), Igh-1b (BD Pharmingen, 553504), CD244.2 (BD Pharmingen, 553533), β2 microglobulin (555299). After washing, the samples were further incubated with avidin-labelled horseradish peroxidase (Life Technologies, 43-4423) at 37 °C for 1 h. After incubating in the dark with ortho-phenylenediamine (Sigma Aldrich, P9029) for 30 min at room temperature(15–25 °C), the reaction was stopped with the addition of 1.5 N H_2_SO_4_. The samples were analysed on a microplate reader (VERSAmax, Molecular Devices) at 492 nm.

### Sample collection and DNA sequencing

Samples from the Korl, Crl, and Tac lines of the C57BL/6N strain were obtained from the National Institute of Food and Drug Safety Evaluation, Korea. Genomic DNA was extracted from the tail tissues of each mouse. Quality assessment of the DNA was performed based on the fluorescence concentration (≥50 ng/μL and total ≥3 μg) using the Quant-iT BR assay kit (Invitrogen), ultraviolet absorbance (OD_260/280_ ≥ 1.7 and OD_260/230_ ≥ 1.5) using a Tecan F-200 Nanodrop spectrophotometer, and degradation assessment on 1% agarose gels (major band of over 10 kb on the gel).

From the DNA samples that met the above quality assessment criteria, 1 μg gDNA was randomly sheared using the Covaris System. The TruSeq DNA PCR-free system (Illumina) was used for library construction following the manufacturer guidelines. Whole-genome sequencing was performed using the Illumina Hiseq. 4000 platform. In addition, we downloaded the public whole-genome sequence data of three C57BL/6N samples from the National Center for Biotechnology Information Sequence Read Archive database. The sequence quality of raw data was assessed with FastQC software^13^, and Trimmomatic-0.33^14^ was used to eliminate potential adapter sequences and low-quality bases.

### Whole-genome re-sequencing and variant calling

The paired-end reads were aligned to the *Mus musculus* reference genome (GRCm38) using Bowtie2^15^ with default settings. Potential PCR duplicates were filtered using the “MarkDuplicates” option in Picard tools (http://picard.sourceforge.net). We then used SAMtools^16^ to construct index files for reference and bam files. Local realignment of sequence reads was performed to correct for any misalignment caused by small insertions and deletions using Genome Analysis Toolkit (GATK) 3.4–46^17^. Base quality score recalibration was performed to obtain accurate quality scores and to correct for variation in quality due to the machine cycle and sequence errors. For variant calling, the “UnifiedGenotyper” and “SelectVariants” arguments in GATK were used and the following criteria were adopted to avoid possible false positives: (1) single nucleotide polymorphisms (SNPs) with a Phred-scaled quality score <30; (2) SNPs with a total count across all samples of mapping quality zero reads (MQ0) ≥4 and “MQ0/(1.0 * DP) >0.1”, where DP stands for read depth; (3) SNPs with FS values (Phred-scaled P-values using Fisher’s exact test) of more than 200 were filtered out to reduce false-positive calls due to strand bias. After discovering whole-genome variants, each variant was annotated using snpEff^18^.

### Population structure analysis

The sequence variation data of 36 mouse strains in the Mouse Genome Project from the Sanger Institute were used to compare the population structures among various strains. Using the whole SNP sets defined from 13 mice, principal components analysis was then performed to detect population stratification in the C57BL/6N strains using SNPRelate^19^. SNPRelate was also used to construct a phylogenetic tree of the samples based on identical-by-state (IBS) methods and to estimate the identical-by-descent (IBD) coefficients to identify the degree of relatedness between each pair of samples.

### Identification of strain-specific enriched variants

To identify the breed-specific homozygous or heterozygous SNPs, enrichment analysis was performed using Snpsift. For statistical evaluation of each SNP’s genotype count data, 2 × 2 contingency tables were constructed, including strain information (Korl versus the others) and an allelic model of homo/heterozygous genotype as the two factors. We performed the Fisher-exact test for each 2 × 2 contingency table. Because of the large multiple testing burden (total of 718,440 SNPs were tested), we adjusted the P-values using FDR correction to control the family-wise error rate at the 0.05 level.

### Detection of selective sweep regions

To detect genome-wide selective sweep signals, genetic differentiation (F_ST_) and nucleotide divergence (*θ*_*π*_) statistics were calculated using the output options in VCFtools^20^ with a sliding window approach (100-kb bin size, 10-kb step size). The *θ*_*π*_ ratio (*θ*_*π*,*other*_/*θ*_*π*,*Korl*_) was calculated as pairwise nucleotide variation.

### Linkage disequilibrium (LD) analysis

Using whole-genome SNP data, the genome-wide LD was estimated by calculating the squared correlation coefficient (r^2^) between all pairs of SNPs with a distance of less than 100 kb between SNPs to analyse the difference of LD patterns between Korl and the other mouse strains using plink2 software^21^. The same method was used to estimate the average LD of each of the bins identified as selective sweep regions.

### General features of Korl and statistical analysis of phenotypes

To analyse the general features of the Korl strain, the phenotypes of Korl mice were compared with those of five C57BL/6 substrains (C57BL/6JJmsSlc, C57BL/6NCrl, C57BL/6NHsd, C57BL/6NTac, and C57BL/6NCrSlc) at 16 weeks of age. To obtain blood samples, the mice were fasted overnight and were induced in a deep state of anaesthesia with isoflurane. For evaluation of haematological parameters, the collected blood was placed in an ethylenediaminetetraacetic acid-containing tube and measured with a haematology analyser (ADVIA 2120i, SIEMENS). To analyse biochemical parameters, the blood was placed in a serum-separation tube and centrifuged at 3,000 rpm for 10 min. Sera were measured with a blood chemistry analyser (HITACHI 7100, HITACHI).

Body weight of mouse substrains was statistically compared using two-way repeated-measures analysis of variance (with significance judged at a P-value < 0.05). In addition, pairwise t-tests with the pooled standard deviation were conducted to compare the difference among the phenotypes (related to length, organ weight, haematology, and blood chemistry) of mouse substrains. All statistical analyses were performed using R.

## Results

### Strain identification and phenotype investigation of Korl mice

**Figure 1:**
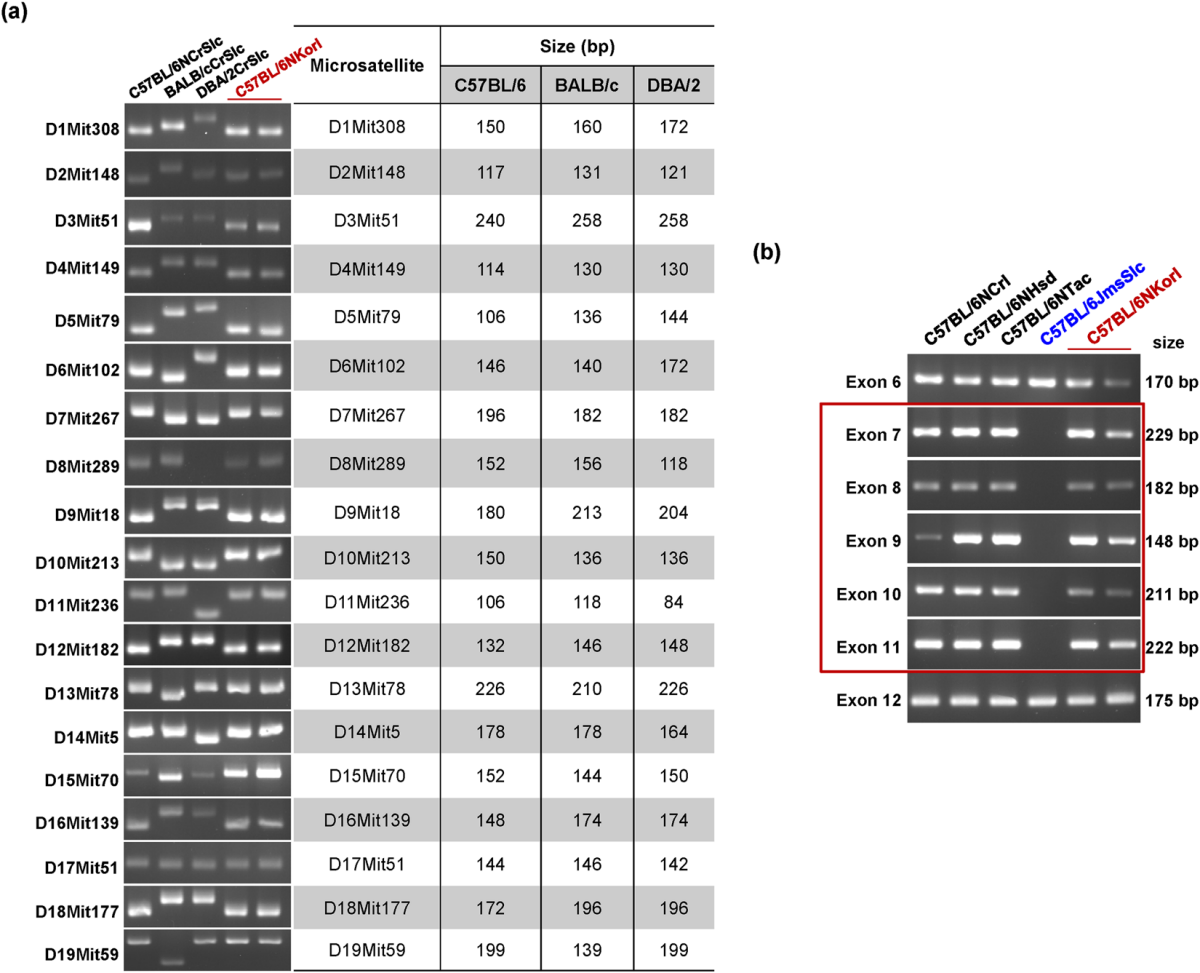
Strain Identification using (**a**) microsatellites and (**b**) genomic PCR specific for exons 6–12 in the *Nnt* gene.

For strain identification of Korl mice before conducting the genome-wide investigation, genetic markers known to be specific to the C57BL/6 or C57BL/6N strain were used. First, 19 microsatellites and MHC types that are genetic markers for the C57BL/6 strain were investigated. All 19 microsatellites and MHC types of Korl mice were identical with those of C57BL/6 mice (Fig. 1a and Supplementary Table S3). These results confirmed that Korl belongs to the C57BL/6 strain.

The PCR results showed no deletion of exons 7–11 of the *Nnt* gene in Korl, which confirmed that Korl belongs to the C57BL/6N and not the C57BL/6J strain (Fig. 1b). Overall, we ascertained that the Korl strain is a substrain of C57BL/6N.

**Figure 2:**
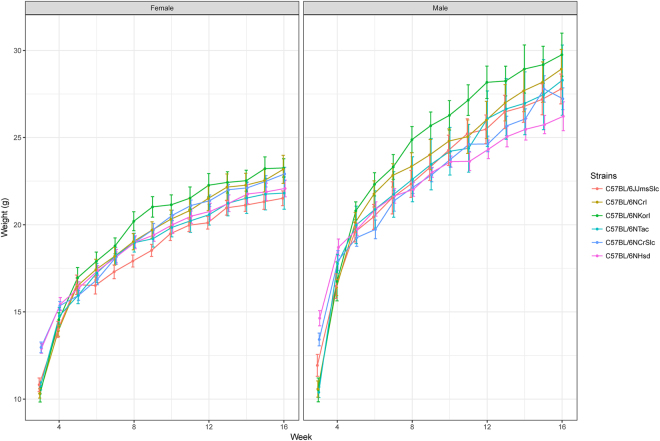
Body weight curve of mouse substrains from the 6^th^ to 16^th^ week.

Comparison of the phenotypic characteristics of Korl with those of the five C57BL/6 substrains showed that the majority of the phenotypes were almost identical among strains with insignificant differences (Table S4), whereas body weight was significantly higher in the Korl mice compared to that of the other strains (P < 0.05; Fig. 2).

### Sequence alignment and variant calling

The whole genomes of six Korl mice (belonging to the 33^rd^ generation), two Crl mice, and two Tac mice were sequenced with a depth of coverage ranging from 36× to 40× (according to the 3-Gb mouse reference genome GRCm38). Produced reads and downloaded public data (see Supplementary Table S5) were aligned to the mouse reference genome to cover about 97% of the genome using Bowtie2, and the detailed statistics of the mapping results are shown in in Supplementary Table S6. We identified 718,440 SNPs and 80,231 InDels (48,694 insertions and 31,537 deletions). Among the 718,440 SNPs, 187,600 (26.1%) were defined in a previous mouse genome re-sequencing study^22^. However, 530,840 SNPs (73.9%) were newly defined in this study as candidates for novel variants in C57BL/6N strains. The detailed results of variant calling and annotation are shown in Supplementary Tables S7 and S8.

### Population differentiation of Korl

**Figure 3:**
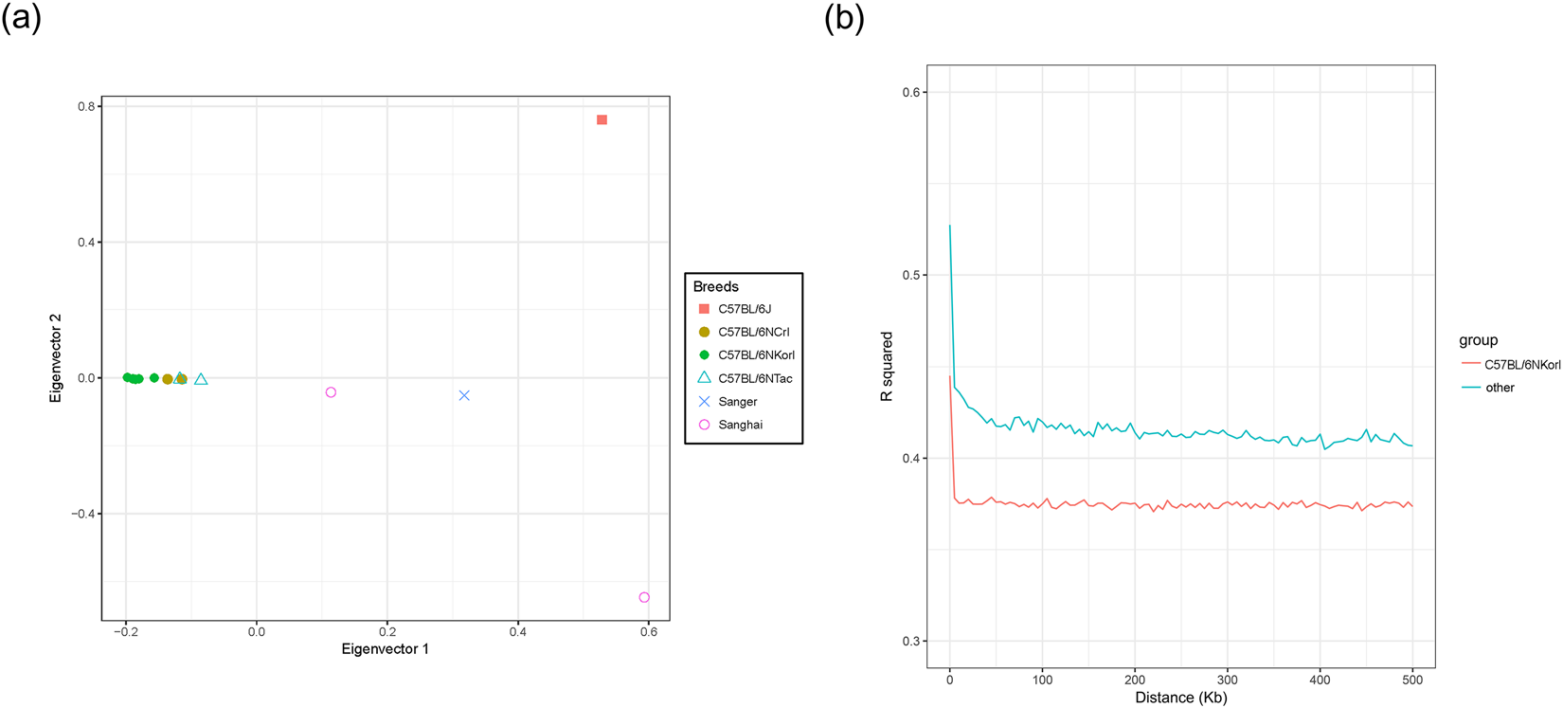
Population differentiation of C57BL/6 mice. **(a)** Principal components analysis of 13 C57BL/6N and C57BL/6J mice. **(b)** Linkage disequilibrium pattern of Korl and other C57BL/6N samples.

Based on the strain identity of Korl, we performed population analyses using whole SNPs identified from 13 mice (see Supplementary Table S5) and the C57BL/6J mouse public data (DRR023979) to determine the population differentiation structure and relationships among substrains of C57BL/6N. In the principal components analysis, there was clear separation between the C57BL/6J and C57BL/6N samples. In addition, samples of the Korl strain were clustered independently and structural differences between populations were revealed along the first and second principal component axes, accounting for 16.69% and 11.31% of the total variance, respectively (Fig. 3a). Moreover, IBS and IBD analyses (Supplementary Fig. S1) also showed clear differences of the Korl samples from the others. Overall, these results indicated that the Korl strain’s genetic delineation is significantly distinct from that of other C57BL/6N strains.

### Identification of strain-specific enriched variants

Statistical analysis revealed loci at which only Korl had distinct genotypes. A total of 4,100 SNPs were identified as Korl-specific SNPs in the statistical test determined based on the number of non-reference genotypes (FDR-adjusted P-value < 0.05). Supplementary Fig. S2 shows the genotype of each sample at significantly different loci, and the majority of these significant SNPs were detected at homozygous non-reference sites.

To investigate the functional characteristics of these SNPs, their closest genes were annotated and subjected to over-representation analysis of gene ontology (GO) (Bonferroni corrected P-value < 0.05). Supplementary Table S9 shows the terms that met the significance threshold in the biological process, cellular components, and molecular function GO categories, and the three terms enriched in the Kyoto Encyclopedia of Genes and Genomes pathway. Among these terms, over-representations of protein binding (GO:0005515), membrane (GO:0016020), and cytoplasm (GO:0005737) were closely related to interaction and transduction of cells with the extracellular environment. In particular, over-representations related to calcium ions (GO:0005516, GO:0005509, mmu04020) were identified, which is known to play a pivotal role in cellular regulation and energy generation. We further conducted in-depth analysis of the exonic regions where the 4,100 SNPs identified are located, which might directly influence the observed increased body weight of the Korl strain. As a result, nine SNPs were found to be located in genes related to an increase of body weight (Table 1).

**Table 1:** Candidate genes affecting the increased body weight in Korl mice.

Gene Symbol	Chr	Start	End	MP ID	Term
**Genes involving Korl-specific SNPs**					
*Bmpr2*	1	59763400	59879014	MP:0001260	increased body weight
*Pmch*	10	88091072	88092375	MP:0001262	decreased body weight
*Ppm1d*	11	85311244	85347066	MP:0001265	decreased body size
*Synj2*	17	5941280	6044290	MP:0001260	increased body weight
*Afg3l2*	18	67404760	67449136	MP:0001265	decreased body size
				MP:0001262	decreased body weight
*Shc1*	3	89418443	89430015	MP:0001262	decreased body weight
				MP:0005378	growth/size/body region phenotype
*Kit*	5	75574916	75656722	MP:0005535	abnormal body temperature
				MP:0005378	growth/size/body region phenotype
*C2cd3*	7	100372233	100470152	MP:0003885	abnormal rostral-caudal body axis extension
*Smg1*	7	118131308	118243670	MP:0001257	increased body length
				MP:0001264	increased body size
				MP:0001260	increased body weight
**Genes involved in selective sweep analysis**					
*Plac9a*	14	25887932	25903100	MP:0010024	increased total body fat amount
*Dusp1*	17	26505590	26508519	MP:0001262	decreased body weight
				MP:0010025	decreased total body fat amount
*Stag3*	5	138280240	138312393	MP:0003960	increased lean body mass

### LD and selective sweep of Korl mice

To obtain more comprehensive information on the genetic profile of Korl mice, genome-wide LD analysis was conducted and compared between Korl and the other strains over increasing distances across the two groups (Fig. 3c). Compared to the other strains, Korl showed much lower LD overall, with differences in certain aspects of LD between the two groups. This analysis revealed the inflow of gene pools during the development of the Korl strain.

**Figure 4:**
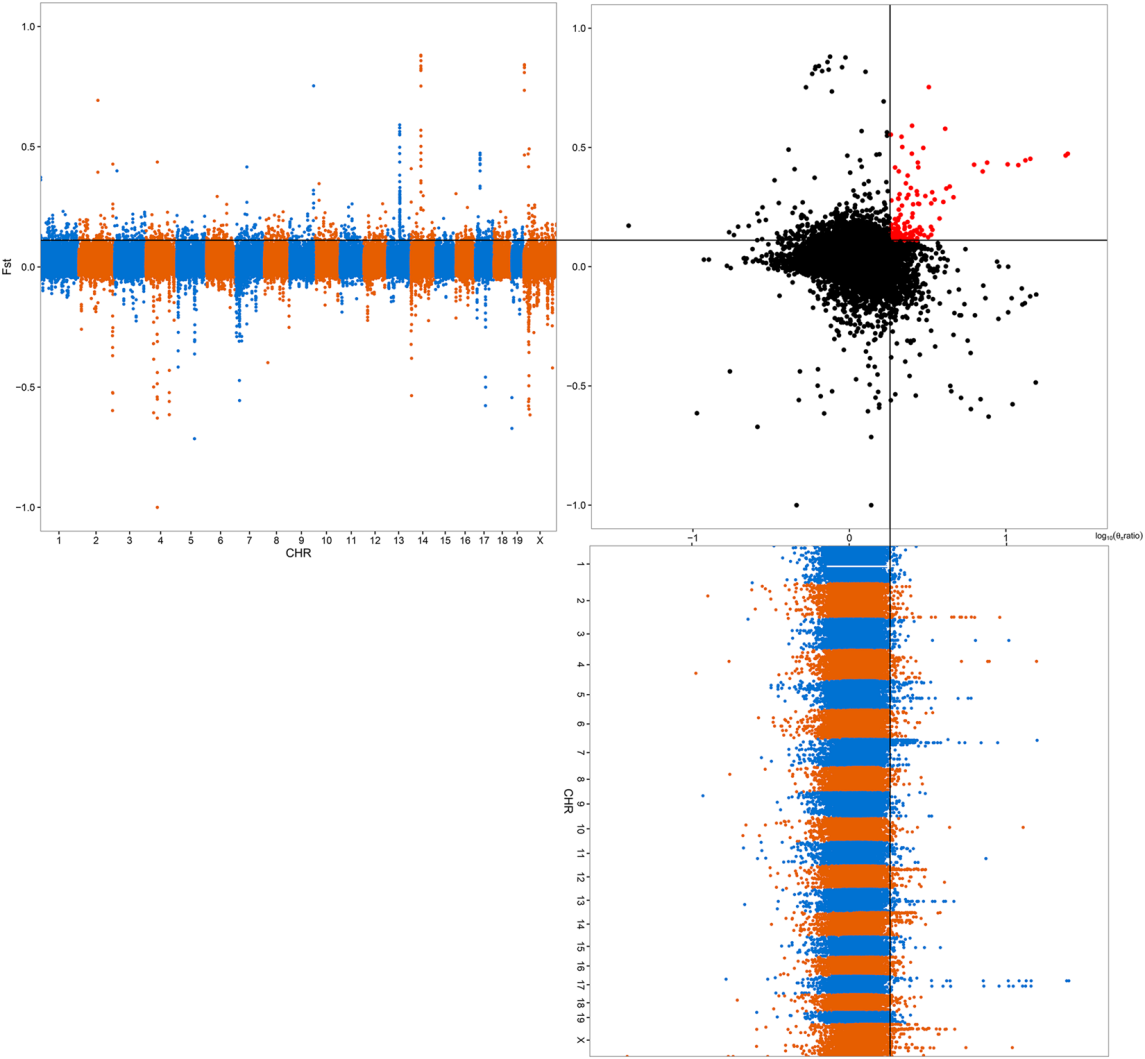
Genomic regions with selective sweep signals in the Korl strain.

To determine the specific genes involved in this introgression, we compared the genomes of the mouse strains to identify within-Korl strain signatures of positive selection. We used the genetic differentiation (Weir and Cockerham’s F_ST_) and pairwise nucleotide variation [log_10_ (*θ*_*π*_ ratio)] statistics to detect candidate genomic regions as significant regions according to previously reported methods^23,24^. To detect reliable results and overcome the limitation of the small sample size, only regions belonging to the top 1% for the two statistics [F_ST_ > 0.11, log_10_ (*θ*_*π*_ ratio) > 0.26] were selected. Consequently, we detected 145 regions that were predicted to be under strong selective pressure (Fig. 4).

Among a total of 218 genes located within the 145 candidate selective sweep regions (see Supplementary Data S2), gene segments of T cell receptor alpha variable (*Trav*) and immunoglobulin kappa chain variable (*Igkv*) were identified in chromosome 14 and 6, respectively. In addition, three genes were identified to play a critical role in body weight based on mammalian phenotype ontology and the literature (Table 1).

## Discussion

We here provide the first report of the genetic profiles of the newly developed C57BL/6N mouse strain Korl. Conventionally, there are two basic requirements for the establishment of an inbred strain: more than 20 consecutive generations of full-sib mating, and all members must be derived from a single breeding pair^25^. Korl, as a substrain of C57BL/6N developed by intensive inbreeding over ~33 generations, fulfils both of these conditions. Furthermore, Korl shows distinct genetic and phenotypic characteristics from other C57BL/6N strains.

One of these unique genetic characteristics was supported by genomic analyses. Principal components, IBS/IBD, and LD analyses conducted at the genome-wide level showed clear division of Korl samples from the other strains. In particular, pairwise relationships obviously demonstrated the existence of an identical region inherited from a common ancestor in the Korl samples. This clear division implies that genetic inflow in the Korl strain has been maintained since its development. In addition, the patterns of population differentiation indicated that Korl belongs to C57BL/6N, but were distinguished from substrains within this group, which is consistent with the results of experiments using genetic markers.

The genomic variants specific for Korl were identified as well as distinctive genome-wide patterns. In particular, anomalistically consecutive missing variants were detected in chromosome 14. Because missing genotypes were found exclusively in all of the Korl samples, the possibility that this was due to a simple technical sequencing error can be ruled out. In recent studies, invariably missing genotypes could be explained by structural variations that prevent intact sequencing^26,27^. Therefore, regions of high-missing genotypes in chromosome 14 would be indicative of a Korl-specific genomic region with unusual structural variation.

With respect to phenotypic characteristics, Korl mice show a significantly higher body weight than the other strains. In our analysis, this phenotypic difference was identified to be caused by the unique genetic background of Korl. GO analysis showed that the regions related to Korl-specific SNPs were fairly closely related to the cell’s direct interaction with the extracellular environment, such as protein binding (GO:0005515), cell adhesion (GO:0007155), and signal transduction (GO:0035556, mmu04020). Although determining the precise mechanisms contributing to this effect is beyond the scope of the present work, this binding has been shown to induce conformational changes with relevance to the development of multicellular organisms^28^. These significant GO categories showed that Korl-specific variants may have contributed to the unique morphological features of this strain, such as the relatively high body weight. Moreover, we identified gene segments related to immunoglobulin (such as *Trav* and *Igkv*). These gene segments are categorized in the immunoglobulin V-set domain (IPR013106), which are immunoglobulin-like domains resembling the antibody variable domain. Immunoglobulin-like domains are known to be involved in various functions such as cell-cell recognition, cell-surface receptors, and the immune system^29,30^. It is expected that this selective sweep region could potentially result in specific immune-related properties of this strain, which requires further investigation.

Since Korl mice have been sib-mated since 2005, the healthiest individuals in each generation were selected as representatives to carry on the strain line. Thereby, a health-related gene pool might have been inherited throughout the generations owing to this unintended artificial selection, which results in incomplete random mating. This could be another factor contributing to the appearance of the increased body weight of Korl mice. Indeed, a genetic cause for this body weight difference was revealed in our whole-genome data based on previously identified features of gene-phenotype relationships in mammals. Therefore, the Korl strain has distinctive features, especially with respect to body weight, and indirect evidence for this increase of body weight was revealed at the genomic level.

## Conclusion

In this study, we determined the unique features of the Korl mouse strain as well as candidate genetic features responsible for its distinct phenotypic characteristics through phenotype experiments and whole-genome re-sequencing. These results suggest that the Korl strain could be a useful resource available to broad research fields requiring mouse models. Furthermore, as the mouse is a suitable model animal for analyses of various human traits and conditions, further profiling of the genetic basis underlying these distinct characteristics and comparative genomic analyses with other substrains may provide new insight into the causes responsible for various traits of interest.

## Electronic supplementary material

Dataset 1

Dataset 2

Supplementary Information
