# Supplementary material for: Genetic and phenotypic characterization of the novel mouse substrain C57BL/6N Korl with increased body weight
Source: Sci Rep. 2017 Oct 27;7:14217. doi: 10.1038/s41598-017-14196-0 (PMC5660189; doi:10.1038/s41598-017-14196-0)

**Supplementary Information for**

**Genetic and phenotypic characterization of the novel mouse substrain C57BL/6N Korl with increased body weight**

Kyung-Min Choi^1,†^, Jaehoon Jung^2,4,†^, Young Min Cho^1^, Kwondo Kim^2,3^, Mi-Gyeong Kim^1^, Jinho Kim^1^, Huibae Kim^1^, Hee Jung Shin^1^, Hae Deun Kim^1^, Seung-Tae Chung^1^, Seoae Cho^2^ and Myeon-Woo Chung^1,*^

^1^Laboratory Animal Resources Division, Toxicological Evaluation and Research Department, National Institute of Food and Drug Safety Evaluation, Cheongju, 28159 Republic of Korea

^2^C&K Genomics, 26 Beobwon-ro 9-gil Bldg. C, #1008 (H business park) Songpa-gu, Seoul 05836, Republic of Korea.

^3^Interdisciplinary Program in Bioinformatics, Seoul National University, Kwan-ak St. 599, Kwan-ak Gu, Seoul, South Korea 151-741, Republic of Korea

^4^Department of Agricultural Biotechnology, Seoul National University, Kwan-ak St. 599, Kwan-ak Gu, Seoul 151-742, Republic of Korea

^*^Corresponding Author Myeon-Woo Chung

(Tel: 043-719-5501, Fax: 043-719-5500, E-mail: mwchung@korea.kr)

^†^This authors contributed equally to this work.

**Table of contents**

**Supplementary Tables S1-S7**

Supplementary Table S1 | Primer information of microsatellites and expected PCR size

Supplementary Table S2 | Primer information for Nnt gene and expected PCR size

Supplementary Table S3 | Major Histocompatibility Complex (MHC) types of C57BL/Korl and C57BL/6

Supplementary Table S4 | Various phenotypes of C57BL/6NKorl

Supplementary Table S5 | Description of samples used in the paper

Supplementary Table S6 | Summary statistics of sequence read mapping results using Bowtie2

Supplementary Table S7 | Detailed called variants in each chromosome

Supplementary Table S8 | Summary results of genetic variants annotation using Snpeff

Supplementary Table S9 | Over-representation of Gene Ontology of Genes related to Korl-specific SNP

**Supplementary Fig. S1-S2**

Supplementary Fig. S1 | Pairwise relationship between each sample using (a) Identical by State (IBS) and (b) Identical by Descent (IBD) Coefficient.

Supplementary Fig. S2 | Genotypic aspects of variants identified in 13 C57BL/6N by whole genome sequencing. Color key: Red – homozygous variants, Beige – heterozygous variants, Aqua blue – reference homozygous sequence, Deep blue – missing genotype

**Table S1. Primer information of microsatellites and expected PCR size.**

| **Microsatellites** | **Primers** | **Size (bp)** | | |
| --- | --- | --- | --- | --- |
|  |  | **C57BL/6** | **BALB/c** | **DBA/2** |
| D1Mit308 | 5' GAGGCTATGAGTCAAATGGACC 3' 5' TTTATGAGGTGCTGAGATGCA 3' | 150 | 160 | 172 |
| D2Mit148 | 5' GTTCTCTGATCTACGGGCATG 3' 5' TTCACTTCTACAAGTTCTACAAGTTCC 3' | 117 | 131 | 121 |
| D3Mit51 | 5' GGCACTGATAGCAGGCCTAG 3' 5' TCTCTTCTGGTATTTCCTTCCG 3' | 240 | 258 | 258 |
| D4Mit149 | 5' TGAATTCAGAAGGATGTGTGTATG 3' ATGTGAGAATCAACACCTGAGG 3' | 114 | 130 | 130 |
| D5Mit79 | 5' ATGCTAAAAAAAAGGCTAAGTTCA 3' 5' CAGTAAATAGCAGGTGTTCATGG 3' | 106 | 136 | 144 |
| D6Mit102 | 5' CCATGTGGATATCTTCCCTTG 3' 5' GTATACCCAGTTGTAAATCTTGTGTG 3' | 146 | 140 | 172 |
| D7Mit267 | 5' CTCTTTCTGTTACATGGTTAGATTTCC 3' 5' AAAGACAGTTGAAGTTGACTTCTGG 3' | 196 | 182 | 182 |
| D8Mit289 | 5' AAAAAGAAAAGAAGGCTTAGTAATGTG 3' 5' CTTGCTATTCATTGCAAAATTCC 3' | 152 | 156 | 118 |
| D9Mit18 | 5' TCACTGTAGCCCAGAGCAGT 3' 5' CCTGTTGTCAACACCTGATG 3' | 180 | 213 | 204 |
| D10Mit213 | 5' CTCCTCCTACTGATTGTCCCC 3' 5' GGGACAAACTTTTAAAAATTGCA 3' | 150 | 136 | 136 |
| D11Mit236 | 5' TGCCACTTCTTTAATACATGCG 3' 5' AATTTCCTTCTACTCCTCTCTGAGC 3' | 106 | 118 | 84 |
| D12Mit182 | 5' GTACATACAATACATCACACAAACGG 3'  5' GGCAAGAAAACAGACCAATAGG 3' | 132 | 146 | 148 |
| D13Mit78 | 5' ACAGCACGGGTTTATCATCC 3' 5' TATGCCTGCCAGGCTTCTAT 3' | 226 | 210 | 226 |
| D14Mit5 | 5' CACATGAACAGAGGGGCAG 3' 5' GTCATGAAGTGCCCACCTTT 3' | 178 | 178 | 164 |
| D15Mit70 | 5'CATTGAGGGTTTGTAGGTTGG 3' 5' ACCCCTGCAAGTTGTCTTTG 3' | 152 | 144 | 150 |
| D16Mit139 | 5' GTATGTAAGGAATGGTCAAATTCTTG 3' 5' TCATTGTGATTGTGAAAGAATGC 3' | 148 | 174 | 174 |
| D17Mit351 | 5' TCTGCCCTGTAACAGGAGCT 3' 5' CTTCTGGAATCAGAGGATCCC 3' | 144 | 146 | 142 |
| D18Mit177 | 5' CTGTAGTTTATCAGTTCACCCTGTG 3' 5' TGTGCTGTTAAACAAATATCTCTGG 3' | 172 | 196 | 196 |
| D19Mit59 | 5' CTCTAACTATCCTCTGACCTTCACA 3' 5' TTTTAAGCAGAACATTGAGGACC 3' | 199 | 139 | 199 |

Table S2. Primer information for Nnt gene and expected PCR size

| ***Nut* gene** | **Primers** | **Size (bp)** |
| --- | --- | --- |
|  |  |  |
| Exon 1 | 5' TCAACTTCACCGTGTGTTC 3' 5' GCAGTGAGCAACACACCACT 3' | **242** |
| Exon 6 | 5' GGGTTTCGATTGCTGTCATT 3' 5' AGTCAGCAGCACTCCTCCAT 3' | **170** |
| Exon 7 | 5' ATTTAGCTGCTGAGGCTGGA 3' 5' GACAAAGACCCGAGAGCAC 3' | **229** |
| Exon 8 | 5' CCAGGCGAGCACTCTCTATT 3' 5' CAGGGTCACAGGAGAACACA 3' | **182** |
| Exon 9 | 5' CCAGCATGCACTCTCTTCTG 3' 5' TGGTCTCCAAGTGCACAGAG 3' | **418** |
| Exon 10 | 5' GACCAATGCCATCTCAGGTT 3' 5' AAGGGCCGACACATTCTATG 3' | **211** |
| Exon 11 | 5' GGCTGCCTTGACTTTGGATA 3' 5' CCTCCTCCTACCTGCAATGT 3' | **222** |
| Exon 12 | 5' GCTGTAGGGCAGTGGTTCTC 3' 5' GGTCACCTAAGGCCATCAGA 3' | **175** |

**Supplementary Table S3.** Major Histocompatibility Complex (MHC) types of C57BL/Korl and C57BL/6

|  | **H-2K** | **H-2D** | **I-A** | **Igh-6** | **Igh-5** | **Igh-4** | **Igh-1** | **CD244** | **ß2 microglobulin** |
| --- | --- | --- | --- | --- | --- | --- | --- | --- | --- |
| **C57BL/6NKorl** | b | b | b | b | b | b | b | 2 | b |
| **C57BL/6** | b | b | b | b | b | b | b | 2 | b |

**Supplementary Table S4.** Various phenotypes of C57BL/6NKorl

| **Group** | **Trait** | **Description** | **male** | | | **Female** | | |
| --- | --- | --- | --- | --- | --- | --- | --- | --- |
|  |  |  | **Sample Size** | **Mean** | **sd** | **Sample Size** | **Mean** | **sd** |
| Length | Body | Head + Body length | 10 | 95.468 | 2.50876 | 10 | 25.70333 | 0.483529 |
|  | Head | - |  | 25.64 | 0.44627 |  | 91.16111 | 1.606654 |
|  | Tail | - |  | 77.708 | 0.998931 |  | 76.51556 | 1.245071 |
|  | Foot | - |  | 17.02 | 0.172562 |  | 16.93778 | 0.234669 |
| Organ Weight | Brain | - | 28 | 0.5178714 | 0.032268 | 37 | 0.5301459 | 0.037 |
|  | Liver | - |  | 1.242918 | 0.132632 |  | 0.996773 | 0.149959 |
|  | Lung | - |  | 0.1944571 | 0.018668 |  | 0.1787243 | 0.026605 |
|  | Heart | - |  | 0.1439821 | 0.015037 |  | 0.1181595 | 0.010988 |
|  | Thymus | - |  | 0.05374286 | 0.009745 |  | 0.05902703 | 0.009774 |
|  | Kidney | - |  | 0.4550821 | 0.076325 |  | 0.3604973 | 0.04683 |
|  | Kidney(L) | - |  | 0.2222107 | 0.039439 |  | 0.175927 | 0.023579 |
|  | Kidney(R) | - |  | 0.2328714 | 0.038133 |  | 0.1845703 | 0.024849 |
|  | Spleen | - |  | 0.075475 | 0.013024 |  | 0.07800811 | 0.02311 |
|  | Testis(L)/Ovary(L) | - |  | 0.08452143 | 0.005941 |  | 0.00369189 | 0.000938 |
|  | Testis(R)/Ovary(R) | - |  | 0.08590357 | 0.005135 |  | 0.00387027 | 0.001199 |
| Hematology | RBC | red blood cell count  (x106 cells/ul) | 27 | 10.32556 | 0.748893 | 35 | 10.09943 | 0.761276 |
|  | HGB | hemoglobin (g/dL) |  | 14.35556 | 1.311292 |  | 14.62 | 1.033782 |
|  | HCT | Hematocrit (%) |  | 52.87037 | 3.773981 |  | 51.06857 | 3.706415 |
|  | MCV | mean corpuscular volume (fL) |  | 51.22963 | 2.087843 |  | 50.59143 | 1.033376 |
|  | MCH | mean corpuscular hemoglobin (pg) |  | 13.93333 | 1.194217 |  | 14.53714 | 1.292759 |
|  | MCHC | mean corpuscular hemoglobin concentration (g/dL) |  | 27.22222 | 2.391223 |  | 28.71429 | 2.276884 |
|  | RDW | red blood cell distribution width (%) |  | 12.85185 | 0.899303 |  | 12.64 | 1.090655 |
|  | PLT | platelet (x103 cells/ul) |  | 1206.296 | 233.1414 |  | 1007.771 | 145.4899 |
|  | MPV | mean platelet volume (fL) |  | 7.711111 | 1.099417 |  | 7.382857 | 0.782712 |
|  | Reti | reticulocyte (%) |  | 61.55556 | 32.50602 |  | 63.88571 | 29.98899 |
|  | WBC | white blood cell count (x103 cells/ul) |  | 3.409259 | 1.672699 |  | 2.320857 | 0.863387 |
|  | NEUT | neutrophil (%) |  | 15.71111 | 5.30075 |  | 16.08514 | 10.6061 |
|  | LYM | lymphocyte (%) |  | 79.37407 | 5.228222 |  | 79.62286 | 10.74633 |
|  | MONO | monocyte (%) |  | 1.603704 | 0.950094 |  | 0.6714286 | 0.384686 |
|  | EOS | eosinophil (%) |  | 1.681481 | 0.86693 |  | 1.437143 | 0.758902 |
|  | BASO | basophil (%) |  | 0.8259259 | 0.663669 |  | 1.294286 | 1.429509 |
| Blood Chemistry | AST | aspartate (U/L) | 11 | 100.9091 | 23.60277 | 27 | 112.8148 | 60.0897 |
|  | ALT | alanine amino transferase (U/L) |  | 30.09091 | 6.090231 |  | 32.22222 | 15.9092 |
|  | ALP | alkaline phosphatase (U/L) |  | 451.8182 | 45.99743 |  | 516.3704 | 159.5513 |
|  | BUN | bilirubin (mg/dL) |  | 21.65455 | 5.457355 |  | 25.75926 | 3.981038 |
|  | GLU | Glucose (mg/dL) |  | 95.45455 | 16.89002 |  | 100.4444 | 22.86471 |
|  | T-CHO | total cholesterol (mg/dL) |  | 103.9091 | 14.19475 |  | 85.22222 | 19.45475 |
|  | TG | triglyceride (mg/dL) |  | 52.72727 | 20.13996 |  | 25.7037 | 7.750011 |
|  | CA | calcium (mg/dL) |  | 8.870909 | 0.240809 |  | 9.006667 | 0.638935 |
|  | CREA | creatinine (mg/dL) |  | 0.2109091 | 0.025867 |  | 0.25 | 0.039419 |
|  | HDL | high-density lipoprotein-cholesterol (mg/dL) |  | 109.3636 | 47.34823 |  | 52.48148 | 36.06337 |
|  | LDL | low-density lipoprotein-cholesterol (mg/dL) |  | 8.109091 | 2.0226 |  | 11.4963 | 2.829036 |
|  | TP | total protein (g/dL) |  | 5.072727 | 0.249363 |  | 5.355556 | 0.532291 |
|  | ALB | albumin (g/dL) |  | 1.772727 | 0.100905 |  | 1.840741 | 0.167008 |

**Supplementary Table S5.** Description of samples used in the paper

| **Sample Name** | **Sex** | **Instrument** | **Sources From** |
| --- | --- | --- | --- |
| K1M | M | Hiseq 4000 | C57BL/6NKorl |
| K2M | M | Hiseq 4000 | C57BL/6NKorl |
| K3M | M | Hiseq 4000 | C57BL/6NKorl |
| K4F | F | Hiseq 4000 | C57BL/6NKorl |
| K5F | F | Hiseq 4000 | C57BL/6NKorl |
| K6F | F | Hiseq 4000 | C57BL/6NKorl |
| T1M | M | Hiseq 4000 | From Taconic Farm |
| T2F | F | Hiseq 4000 | From Taconic Farm |
| C1M | M | Hiseq 4000 | From Charles River |
| C2F | F | Hiseq 4000 | From Charles River |
| S1 | M | Hiseq X Ten | From Shanghai Institutes for Biological Sciences |
| S2 | F | Hiseq X Ten | From Shanghai Institute for Biological Sciences |
| Sanger | S | Illumina Genome Analyzer 2 | From Sanger Institute |

**Supplementary Table S6**. Summary statistics of sequence read mapping results using Bowtie2

| Sample Name | reads | **overall alignment rate** |
| --- | --- | --- |
| K3M | 354929312 | **97.49%** |
| K5F | 405286659 | **97.52%** |
| K2M | 384762528 | **97.40%** |
| T1M | 425004744 | **97.53%** |
| C2F | 357371864 | **97.42%** |
| T2F | 457952934 | **97.74%** |
| K4F | 362452953 | **97.49%** |
| K1M | 384985183 | **97.66%** |
| C1M | 452822917 | **97.43%** |
| K6F | 456332987 | **97.97%** |
| S1 | 370737216 | **96.99%** |
| S2 | 341532001 | **97.96%** |
| Sanger | 288888313 | **98.51%** |

**Supplementary Table S7**. Detailed called variants in each chromosome

| **Chromosome** | **length** | **variants** | **Variants rate** |
| --- | --- | --- | --- |
| 1 | 195,471,971 | 53,372 | 3,662 |
| 2 | 182,113,224 | 45,502 | 4,002 |
| 3 | 160,039,680 | 41,715 | 3,836 |
| 4 | 156,508,116 | 47,367 | 3,304 |
| 5 | 151,834,684 | 44,753 | 3,392 |
| 6 | 149,736,546 | 38,986 | 3,840 |
| 7 | 145,441,459 | 41,664 | 3,490 |
| 8 | 129,401,213 | 36,104 | 3,584 |
| 9 | 124,595,110 | 31,084 | 4,008 |
| 10 | 130,694,993 | 35,286 | 3,703 |
| 11 | 122,082,543 | 31,554 | 3,869 |
| 12 | 120,129,022 | 32,431 | 3,704 |
| 13 | 120,421,639 | 33,748 | 3,568 |
| 14 | 124,902,244 | 35,175 | 3,550 |
| 15 | 104,043,685 | 27,301 | 3,810 |
| 16 | 98,207,768 | 25,363 | 3,872 |
| 17 | 94,987,271 | 26,540 | 3,579 |
| 18 | 90,702,639 | 22,210 | 4,083 |
| 19 | 61,431,566 | 14,854 | 4,135 |
| X | 171,031,299 | 53,431 | 3,200 |
| Total | 2,633,776,672 | 718,440 | 3665.966 |

**Supplementary Table S8.** Summary results of genetic variants annotation using Snpeff

|  | Tac | | Crl | | Korl | | Shanghai | | Sanger | |
| --- | --- | --- | --- | --- | --- | --- | --- | --- | --- | --- |
|  | SNP | INDEL | SNP | INDEL | SNP | INDEL | SNP | INDEL | SNP | INDEL |
| **Region Type  (permission for repetitive counts)** | | | | | | | | | | |
| DOWNSTREAM | 157,760 | 16,007 | 157,705 | 16,004 | 159,048 | 16,006 | 155,092 | 15,977 | 133,699 | 12,055 |
| EXON | 12,938 | 803 | 12,926 | 803 | 13,146 | 804 | 12,608 | 801 | 11,050 | 667 |
| INTERGENIC | 456,276 | 50,176 | 456,356 | 50,173 | 467,139 | 50,270 | 438,201 | 49,876 | 364,493 | 36,493 |
| INTRON | 834,946 | 94,440 | 834,618 | 94,427 | 838,000 | 94,526 | 829,510 | 94,289 | 720,658 | 71,077 |
| SPLICE_SITE_ACCEPTOR | 178 | 19 | 178 | 19 | 178 | 19 | 178 | 19 | 156 | 16 |
| SPLICE_SITE_DONOR | 123 | 12 | 123 | 12 | 123 | 12 | 123 | 12 | 98 | 12 |
| SPLICE_SITE_REGION | 1,414 | 142 | 1,413 | 139 | 1,431 | 142 | 1,394 | 139 | 1,306 | 120 |
| TRANSCRIPT | 720 | 55 | 721 | 55 | 725 | 55 | 702 | 55 | 618 | 39 |
| UPSTREAM | 178,597 | 19,023 | 178,338 | 19,018 | 180,310 | 19,051 | 175,582 | 18,950 | 146,915 | 14,011 |
| UTR_3_PRIME | 7,831 | 655 | 7,826 | 655 | 7,850 | 655 | 7,800 | 655 | 7,105 | 530 |
| UTR_5_PRIME | 1,898 | 183 | 1,909 | 183 | 1,948 | 185 | 1,859 | 185 | 1,144 | 79 |
| **Functional Class** | | | | | | | | | | |
| MISSENSE | 2957 | - | 2929 | - | 2,979 | - | 2,898 | - | 2,570 | - |
| NONSENSE | 69 | - | 68 | - | 69 | - | 68 | - | 63 | - |
| SILENT | 2003 | - | 2010 | - | 2,034 | - | 1,966 | - | 1,710 | - |
| Total | 712,242 | 80,146 | 711,980 | 80,135 | 717,660 | 80,218 | 704,794 | 80,019 | 607,197 | 59,756 |

**Supplementary Table S9.** Over-representation of Gene Ontology of Genes related to Korl-specific SNP

| **GO ID** | **GO Term** | **Count** | **FDR** |
| --- | --- | --- | --- |
| **BP** | | | |
| GO:0007155 | cell adhesion | 65 | 1.32E-05 |
| GO:0007156 | homophilic cell adhesion via plasma membrane adhesion molecules | 30 | 4.26E-04 |
| GO:0042391 | regulation of membrane potential | 22 | 6.93E-04 |
| GO:0035556 | intracellular signal transduction | 51 | 0.00465 |
| GO:0007416 | synapse assembly | 14 | 0.004984 |
| GO:0007399 | nervous system development | 48 | 0.006145 |
| GO:0007605 | sensory perception of sound | 24 | 0.02275 |
| GO:0034765 | regulation of ion transmembrane transport | 23 | 0.039432 |
| GO:0035418 | protein localization to synapse | 8 | 0.043686 |
| **CC** | | | |
| GO:0045202 | synapse | 80 | 3.38E-11 |
| GO:0016020 | membrane | 552 | 2.21E-09 |
| GO:0030424 | axon | 61 | 1.01E-08 |
| GO:0043025 | neuronal cell body | 75 | 1.03E-07 |
| GO:0014069 | postsynaptic density | 45 | 1.11E-07 |
| GO:0030054 | cell junction | 87 | 3.60E-06 |
| GO:0030425 | dendrite | 67 | 3.66E-06 |
| GO:0043005 | neuron projection | 57 | 8.51E-05 |
| GO:0005737 | cytoplasm | 498 | 2.98E-04 |
| GO:0042995 | cell projection | 79 | 7.67E-04 |
| GO:0060076 | excitatory synapse | 13 | 0.002047 |
| GO:0045211 | postsynaptic membrane | 34 | 0.004275 |
| GO:0005856 | cytoskeleton | 106 | 0.014513 |
| GO:0042734 | presynaptic membrane | 17 | 0.017743 |
| **MF** | | | |
| GO:0005515 | protein binding | 367 | 3.54E-12 |
| GO:0005216 | ion channel activity | 29 | 0.002428 |
| GO:0046872 | metal ion binding | 267 | 0.010318 |
| GO:0005516 | calmodulin binding | 29 | 0.013494 |
| GO:0005509 | calcium ion binding | 74 | 0.01969 |
| **KEGG Pathway** | | | |
| mmu04724 | Glutamatergic synapse | 25 | 1.12E-04 |
| mmu04020 | Calcium signaling pathway | 29 | 0.005503 |
| mmu05412 | Arrhythmogenic right ventricular cardiomyopathy (ARVC) | 16 | 0.02613 |

**Supplementary Figure S1**. Pairwise relationship between each sample using (a) Identical by State (IBS) and (b) Identical by Descent (IBD) Coefficient.


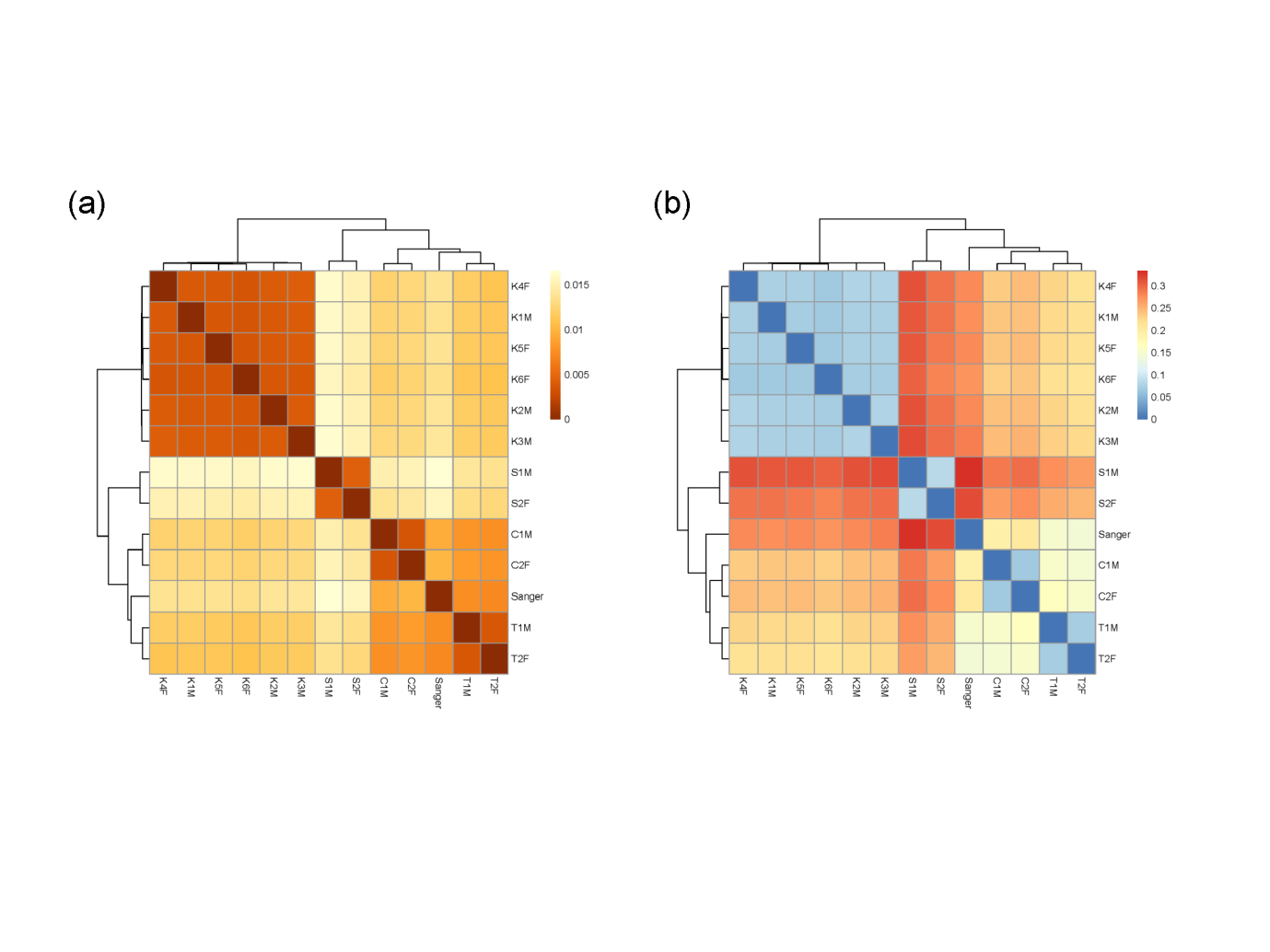


**Supplementary Figure S2**. Genotypic aspects of variants identified in 13 C57BL/6N by whole genome sequencing. Color key: Red – homozygous variants, Beige – heterozygous variants, Aqua blue – reference homozygous sequence, Deep blue – missing genotype


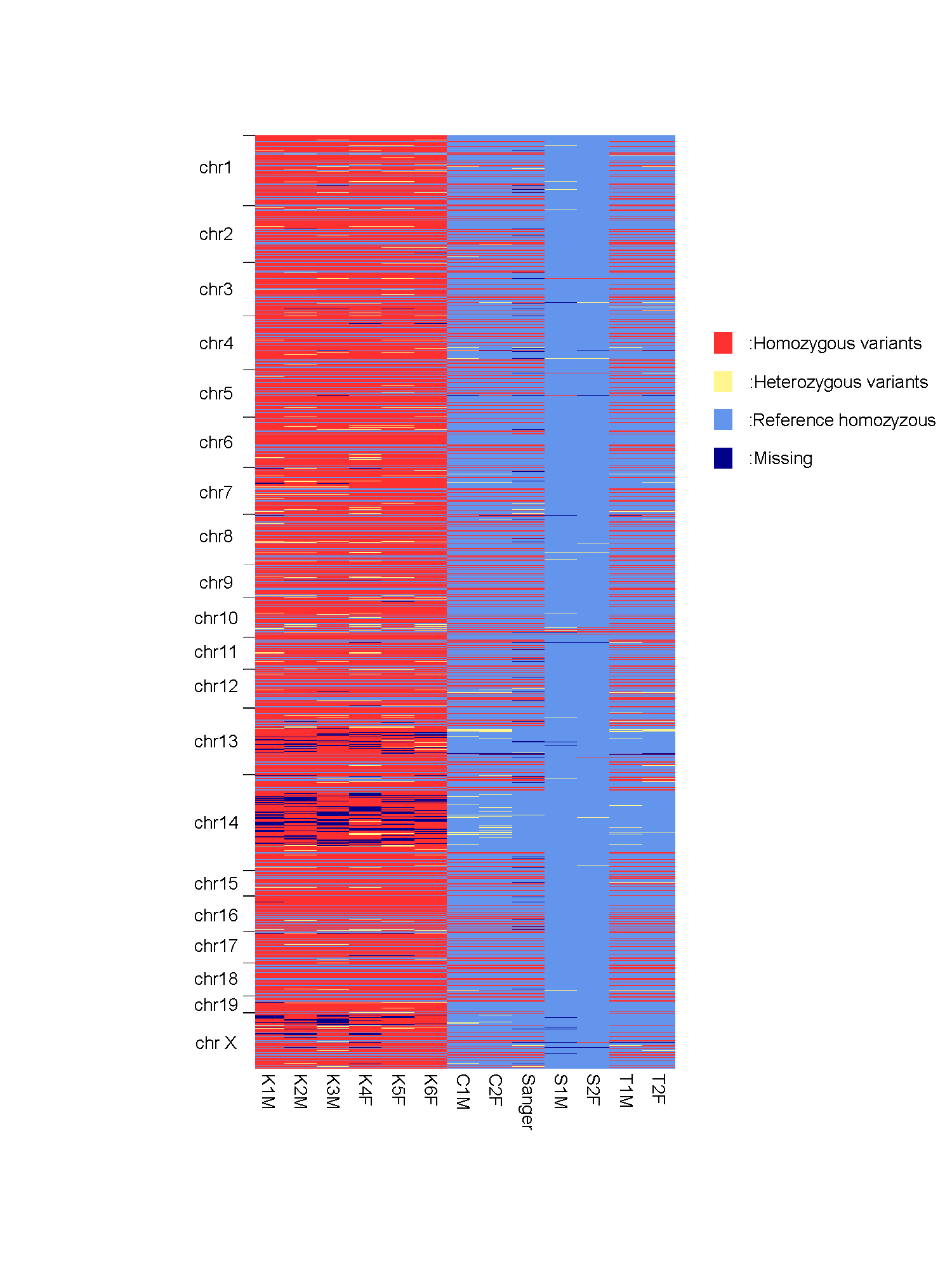

Supplement: Supplementary file 3 — Supplementary Information [file 41598_2017_14196_MOESM3_ESM.docx]
